# Qualitative Assessment of Microalgae–Bacteria Biofilm Development on K5 Carriers: Photoheterotrophic Growth in Wastewater

**DOI:** 10.3390/microorganisms13051060

**Published:** 2025-05-02

**Authors:** Henrique Sousa, Kerry A. Kinney, Cátia A. Sousa, Manuel Simões

**Affiliations:** 1 LEPABE—Laboratory for Process Engineering, Environment, Biotechnology and Energy, Faculty of Engineering, University of Porto, Rua Dr. Roberto Frias, 4200-465 Porto, Portugal; 2 ALiCE—Associate Laboratory in Chemical Engineering, Faculty of Engineering, University of Porto, Rua Dr. Roberto Frias, 4200-465 Porto, Portugal; 3 Architectural, and Environmental Engineering, Department of Civil, University of Texas, 301E E Dean Keeton St. c1700, Austin, TX 78712, USA; 4 ISEP/P.PORTO, School of Engineering, Polytechnic of Porto, Rua Dr. António Bernardino de Almeida, 431, 4249-015 Porto, Portugal; 5 CIETI, Center for Innovation in Engineering and Industrial Technology, School of Engineering, Polytechnic of Porto, Rua Dr. António Bernardino de Almeida, 431, 4249-015 Porto, Portugal

**Keywords:** microalga-bacteria biofilms, profilometry, microbial interactions, wastewater polishing

## Abstract

Wastewater (WW) treatment using biofilms harboring bacteria and microalgae is considered a promising polishing solution to improve current treatment technologies present in wastewater treatment plants (WWTPs), but their interaction in a sessile community remains to be understood. In this work, multi-species biofilms of *Chlorella vulgaris*, *Chlorella sorokiniana*, or *Scenedesmus obliquus* were selected as representative microalgae species of interest for WW bioremediation, and *Rhodococcus fascians*, *Acinetobacter calcoaceticus*, or *Leucobacter* sp. were selected as the bacteria for co-cultivation in a synthetic WW since they are normally found in WW treatment processes. The attached consortia were developed in specific carriers (K5 carriers) for 168 h, and their biofilm formation ability was evaluated in a profilometer and via scanning electron microscopy (SEM) imaging. From the selected microorganisms, *C. sorokiniana* was the microalga that adapted best to co-cultivation with *R. fascians* and *A. calcoaceticus*, developing a thicker biofilm in these two consortia (3.44 ± 0.5 and 4.51 ± 0.8 µm, respectively) in comparison to the respective axenic cultures (2.55 ± 0.7 µm). In contrast, *Leucobacter* sp. did not promote biofilm growth in association with *C. vulgaris* and *C. sorokiniana*, while *S. obliquus* was not disturbed by the presence of this bacterium. Some bacterial clusters were observed through SEM, especially in *A. calcoaceticus* cultures in the presence of microalgae. In some combinations (especially when *C. vulgaris* was co-cultivated with bacteria), the presence of bacteria was able to increase the number of microalga cells adhered to the K5 carrier. This study shows that biofilm development was distinctly dependent on the co-cultivated species, where synergy in biofilm formation was highly dependent on the microalgae and bacteria species. Moreover, profilometry appears to be a promising method for biofilm analyses.

## 1. Introduction

The conventional methods used in wastewater (WW) treatment plants (WWTPs) (such as conventional activated sludge (CAS), ozonation, activated carbon adsorption, membrane filtration, chlorination, and UV treatment) are not entirely practical for WW treatment [1]. On the other side, advanced oxidation methods (such as photocatalysis, electrochemical oxidation, and catalytic wet oxidation) are associated with technological limitations (no full-size applications so far) and with high purchase and maintenance costs [1,2,3]. Microalgae-based systems are garnering interest as a cost-effective and environment-friendly solution for WW bioremediation [4]. Moreover, microbial consortia tend to be more resilient and stable in the face of sudden environmental changes, as well as invasion by predators or competing species, compared to individual species on their own [1,2,3]. As such, multi-species cultures containing microalgae and bacteria have emerged as a promising and sustainable approach for WW bioremediation since these consortia can offer a viable and cost-effective alternative to conventional WW treatment processes, particularly in the tertiary step [1,2,3]. Studies have shown that bacteria can promote microalgal growth by producing growth-promoting hormones, while microalgae release metabolic compounds, such as extracellular polymeric substances (EPS), that bacteria can utilize for their metabolism [4,5]. Additionally, the production of EPS plays a key role in cell-to-cell communication between microalgae and bacteria [6]. These synergistic relationships enhance nutrient recycling, pollutant degradation, and overall system stability, making microalgae–bacteria consortia a sustainable solution for WW treatment [7].

Nevertheless, the specific interactions between strains are not the primary focus of most studies involving biological consortia for WW treatment; instead, they focus on the overall performance of the system. Therefore, the microalgae species selected for this work are commonly found in WW treatment studies, growing in the presence of different microorganisms and having a prominent role in the removal of nutrients and contaminants of emerging concern (CECs) from WW: (i) *Chlorella vulgaris* has been extensively studied for WW polishing [8,9,10]; (ii) *Chlorella sorokiniana* was selected due to its presence in a rotating algal bioreactor (RAB) used for municipal WW treatment and was co-cultivated with bacteria for WW treatment [11,12]; and (iii) *Scenedesmus obliquus* has a remarkable capacity to attach to different surfaces, showing high nutrient removal ability [11,13]. The selected bacteria (*Acinetobacter calcoaceticus*, *Leucobacter* sp., and *Rhodococcus fascians*) have been previously tested in consortia with microalgae and found in WW treatment processes [14,15]. After the selection of the bacterial strains, an assay was done on the ability of each consortium to attach to the surface of K5 carriers using a synthetic WW for 168 h. These carriers are important for bioremediation technologies, as they offer a high surface area for cell attachment, improving the capacity for WW polishing, in contrast to other conventional methods [16]. For that purpose, microorganisms were co-cultivated with the K5 carriers, and the biofilms that had been subsequently developed were analyzed using profilometry and scanning electron microscope (SEM) to characterize the structure of each consortium grown in the presence of WW. This study is the pioneer in the complementary use of profilometry and SEM imaging to characterize selected microalgae and bacteria species for competitive biofilm development to improve WW polishing.

## 2. Materials and Methods

### 2.1. Microorganisms, Culture Medium, and Experimental Setup

*C. vulgaris* AGF002, provided via AllMicroalgae (Pataias, Portugal), was used as a model organism for wastewater bioremediation studies [14,17,18,19]. *C. sorokiniana* UTEX B 3179 was selected because of its isolation source—an RAB municipal WW treatment system. *S. obliquus* UTEX 393 was selected due to the reported ability to develop biofilm-like structures in WWTPs with efficient nutrient removal ability [11,20,21]. These microalgae were maintained on the Organization for Economic Co-operation and Development (OECD) medium with 15 g/L agar [22]. Weekly pre-cultures were prepared by inoculating 40 mL of OECD medium with microalgal cells in 100 mL Erlenmeyer flasks. These were incubated at 25 °C for three days on an orbital shaker at 100 rpm under continuous “cool white” fluorescent light (4000–4200 K, 3.6 cd at the flask surface). Temperature, light intensity, and pH (7.5 ± 0.5) were carefully controlled. The cultures were obtained by inoculating the cells at an initial concentration of 1 × 10^6^ cells/mL from the pre-cultures in 200 mL OECD medium in 500 mL Erlenmeyer flasks. The cultures were incubated for 3–4 days under the conditions described for the pre-cultures. After incubation, the microalgae cells were centrifuged at 4000× *g* for 5 min and resuspended in OECD medium.

The bacteria *R. fascians* and *Leucobacter* sp. were isolated from a *C. vulgaris* photobioreactor and identified using 16S rRNA gene sequencing [14], while *A. calcoaceticus* was isolated from a water system [23]. The bacterial strains were maintained on Tryptic Soy Agar (TSA) medium with 15 g/L agar (VWR©). Weekly pre-cultures were prepared by inoculating 40 mL of Tryptic Soy Broth (TSB) medium with bacterial cells in 100 mL Erlenmeyer flasks [24], followed by incubation at 25 °C for one day on an orbital shaker at 100 rpm. The cultures were incubated for 1 day under the conditions described for the pre-cultures. After incubation, the bacterial cells were centrifuged at 4000× *g* for 5 min and suspended in deionized water.

The microorganisms, in the exponential phase of growth, were inoculated to have a final concentration of 1 × 10^7^ cells/mL for microalgae and Log 4.52 CFU/cm^2^ for bacteria in 250 mL Erlenmeyer flasks at a final volume of the suspension of 125 mL. The microorganisms were co-cultivated on K5 carriers, as described in Section 2.2. The microalgae and bacteria were cultivated in synthetic WW, mimicking a secondary effluent (prepared as described by Gonçalves et al. [2]), and sterilized in an autoclave at 121 °C for 21 min. After 168 h, the carriers were removed and assessed for the development of a dual-species biofilm on the surface of the carrier.

### 2.2. K5 Carriers

The K5 carriers are made of high-density polyethylene (HDPE), selected due to the high surface area in contrast to other carriers available. They were provided by AnoxKaldnes©, with each K5 carrier having a surface area of 2420 mm^2^, 25 mm diameter, and a height of 3.5 mm [25]. These carriers are conventionally used to support microorganisms in moving bed bioreactor systems capable of remediation of highly nitrified WWs or other contaminated water bodies [16]. For this study, the K5 carriers were washed, disinfected, and inserted in the 250 mL Erlenmeyer flasks for biofilm growth, as described in Section 2.1.

### 2.3. Profilometry

The optical profilometer VK-X1100 (Keyence, Itasca, IL, USA), located in Texas Material Instruments at the University of Texas at Austin, used in this work is a laser microscope capable of measuring the roughness and thickness of a film with a nanometer resolution [26]. The device essentially includes a light source and a light-receiving component, an objective lens, a half-mirror, and a pinhole, all to apply the confocal theory to find the peak of the sample [26]. Thus, all the combinations of microalgae and bacteria in single- and dual-species biofilms were assessed in terms of thickness and roughness after 168 h of cultivation on the K5 carriers. Most samples were examined under a 50× magnification, using the optical lens and laser for height and roughness measurement. The approach to assessing roughness, the arithmetic average of the distance, and the standard deviation of the heights, essential for surface characterization, are further detailed [27]. At least three images were taken from each sample at different locations to guarantee the randomness of each image and achieve a more real approximation to the data obtained from each biofilm. The samples were also measured, taking into account the topography of the carrier.

### 2.4. Scanning Electron Microscopy

After 168 h of growth, the microalgae and bacteria biofilms on the K5 carriers were analyzed in an SEM Apreo 2S LoVac (Thermo Fisher Scientific Inc., Waltham, MA, USA), with the use of sputter coater (EMS©, Hatfield, PA, USA) for prior sample preparation (both devices located at the Texas Material Instruments at the University of Texas at Austin). Previous assessments of the samples indicated the need for a coating due to the fast degradation of the biofilms under SEM conditions. The samples were then mounted in adhesive conductive tape and platinum-coated before SEM analysis. High-resolution images were taken under several magnifications to show the distribution of the microorganisms on the surface of the carriers, as well as the interactions between microalgae and bacteria when co-cultivated.

### 2.5. Reproducibility of the Results and Statistical Analysis

All findings were repeatedly tested, independently, at least three times with duplicates (*n* ≥ 6). The statistical analysis was accomplished via a one-way ANOVA, followed by the Tukey–Kramer multiple comparison method. *p* values < 0.05 were considered statistically significant.

## 3. Results and Discussion

The relationship between microalgae and bacteria in sessile structures has been studied in natural ecosystems, mainly in periphyton-type communities [28]. Replicating these types of structures could provide new answers related to their cellular interaction, co-growth, and metabolic interactions for the development of novel and sustainable bioremediation approaches [29]. This work qualitatively assessed selected dual-species biofilm combinations on a specific surface developed for maximum cellular attachment, the K5 carriers, in a synthetic WW, using innovative measurement techniques, specifically profilometry.

**Figure 1:**
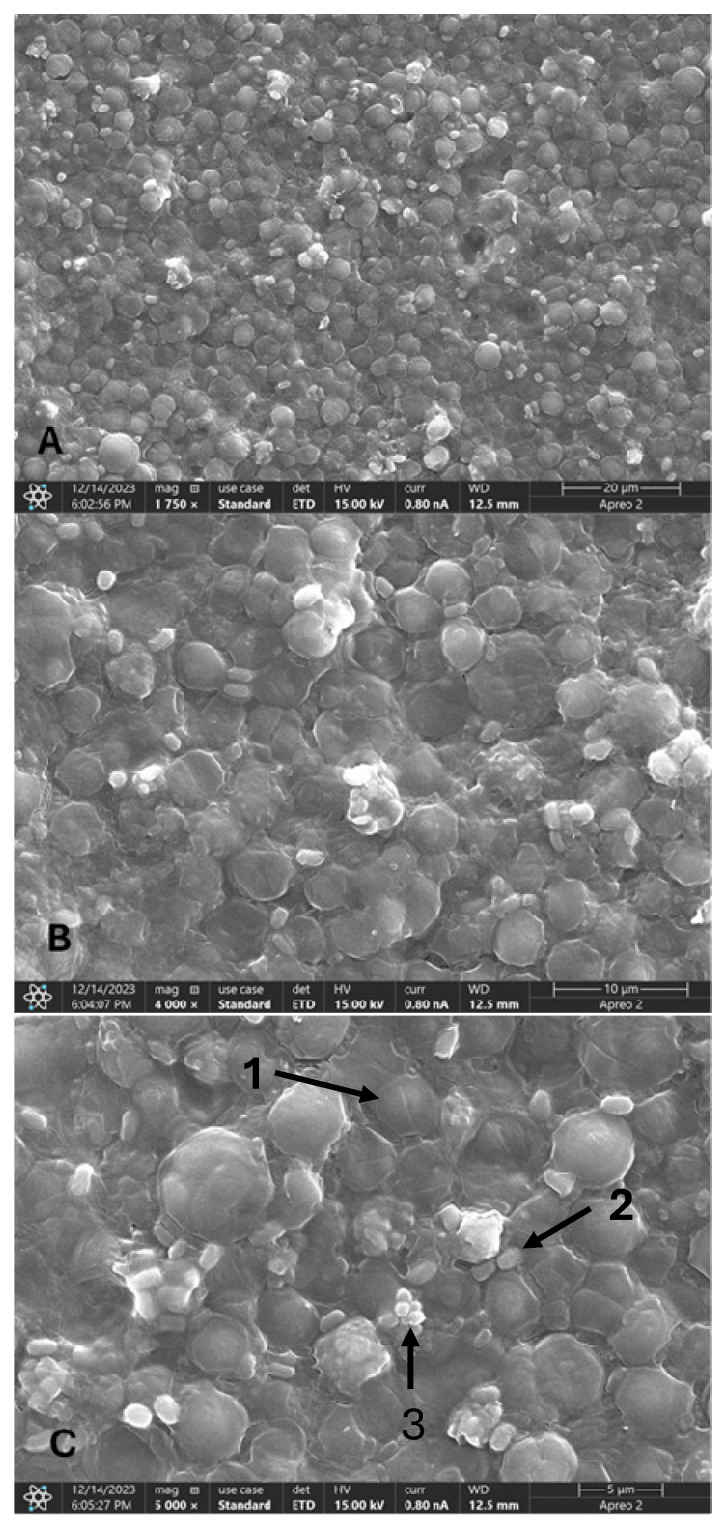
SEM images of *C. sorokiniana* w/*A. calcoaceticus* biofilm at different magnifications: (**A**) 1750×, (**B**) 4000×, and (**C**) 5000×. Arrow 1—*C. sorokiniana* cell. Arrow 2—*A. calcoaceticus* cell. Arrow 3—bacterium cluster.

SEM imaging was used to better understand the distribution of the microorganisms on the surface of the K5 carrier and the biofilm formation at a very high magnification. An example of this imaging is shown in Figure 1, depicting *C. sorokiniana* and *A. calcoaceticus* biofilm at a max 5000× magnification (the remaining SEM images can be found in Supplementary Materials—Figures S1–S8). The C. *sorokiniana* cells, when co-cultivated with *A. calcoaceticus*, cover the most surface in contrast to other consortia, followed by the *S. obliquus* cells co-cultivated with *Leucobacter* sp. *C. vulgaris* cells in all the consortia tested showed the least adhesion potential, although some improvement could be detected when in the presence of *Leucobacter* sp. (Figures S3–S5). From the three microalgae tested, *C. vulgaris* has been highlighted for the ability to attach to different surfaces [30,31]. Irving et al. [31] noticed that introducing bacteria could improve the initial attachment of *C. vulgaris* to surfaces since they can use the EPS produced from other microorganisms to create a cell-dense biofilm. Also, from the SEM images, it is possible to visualize a matrix involving both larger (microalga) and smaller (bacterium) cells in most cases. Also, some bacterial clusters are seen in Figure 1, especially at higher magnifications. This behavior is noticeable in several WW treatment studies, as *Acinetobacter* sp. is recurrently reported to be involved in forming granules [15,32,33]. The SEM images further demonstrate that the presence of bacteria can influence the attachment of microalgae to a specific surface, proposing the existence of synergistic relations related to EPS production.

**Figure 2:**
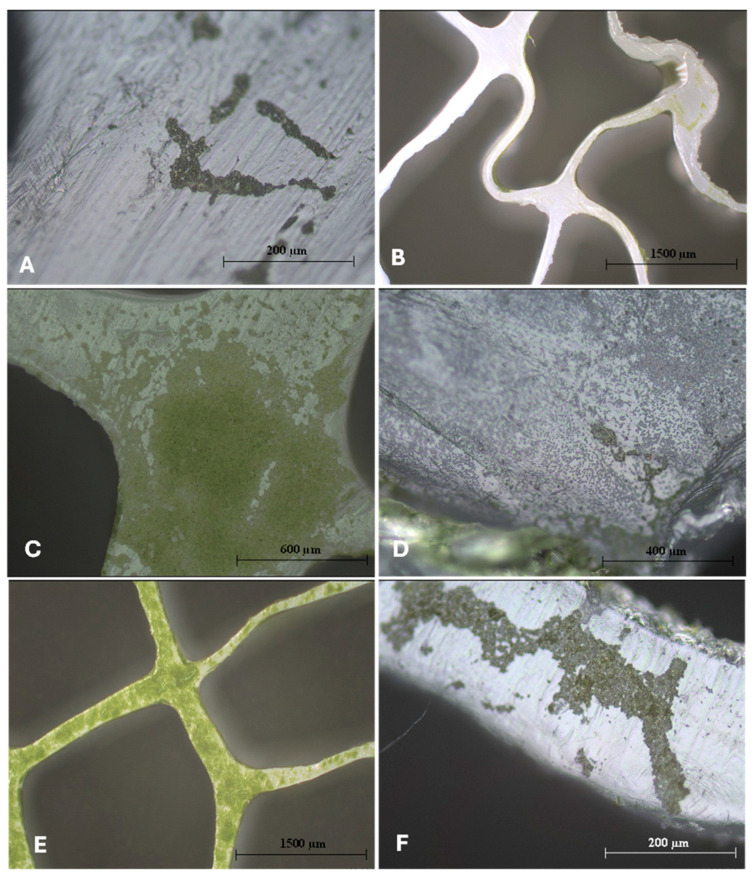
Inspections at different magnifications were taken through a profilometer of the different biofilms studied, showing the cells on the surface of the carrier. The shape of the K5 carrier is well detailed in (**B**,**E**), with irregularities and curves throughout its structure (darker background). (**A**) Axenic *C. vulgaris*. (**B**) *C. vulgaris* w/*R. fascians*. (**C**) *C. sorokiniana* w/*Leucobacter* sp. (**D**) *C. vulgaris* w/*Leucobacter* sp. (**E**) *C. sorokiniana* w/*A. calcoaceticus*. (**F**) Axenic *S. obliquus*.

Additionally, inspections of the dual-species biofilms (as well as some microalgal axenic cultures) were taken with the support of a profilometer and are presented in Figure 2 and the Supplementary Materials, Figure S9. The highest presence of microalgae on the total surface of the carrier was detected in the *C. sorokiniana* cultures (both in the presence of bacteria and the axenic cultures (Figure S9)), followed by *S. obliquus*. On the other hand, the lowest microalgal presence was detected in *C. vulgaris* cultures. From the observations made (both in live observations and SEM images), the accumulation of *C. vulgaris* cells mainly was present in the rougher edges of the carrier and corners, which may suggest a lower surface adhesion than the other microalgae. This microalga has already shown a lower number of cells attached to surfaces than other species [30]. The same study [30] suggested a higher retention time to increase the number of cells in the attached form. Irving et al. [31] also detected a higher cellular density for *S. obliquus* and lower for *C. vulgaris* in different types of surfaces and demonstrated that both microalgae prefer planktonic growth in axenic cultures.

*C. sorokiniana* has also been reported to have an excellent adhesion rate in several environmental conditions [34], even played a prominent role in biofouling formation [35]. Since this specific strain was isolated from an RAB for municipal WW treatment (UTEX B 3179), the biofilm content observed in the images is not surprising. The type of material and surface roughness can also severely affect the cell attachment of microalgae and bacteria [36], which can also explain the differences between the different combinations of the microorganisms tested.

**Figure 3:**
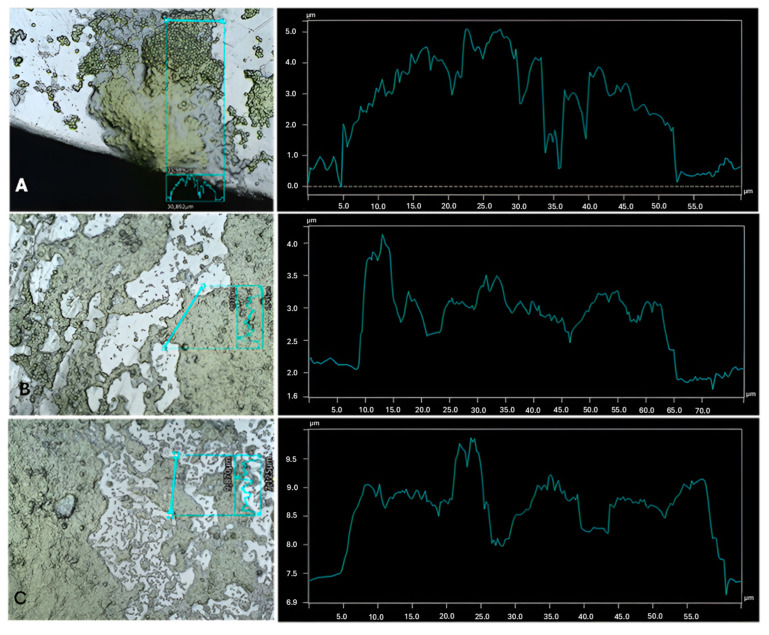
Biofilm profiles for *C. sorokiniana* in co-cultivation with three different bacteria. (**A**) *C. sorokiniana* w/*A. calcoaceticus*. (**B**) *C. sorokiniana* w/*R. fascians*. (**C**) *C. sorokiniana* w/*Leucobacter* sp. The imaging and the topography data were obtained using the profilometer.

**Figure 4:**
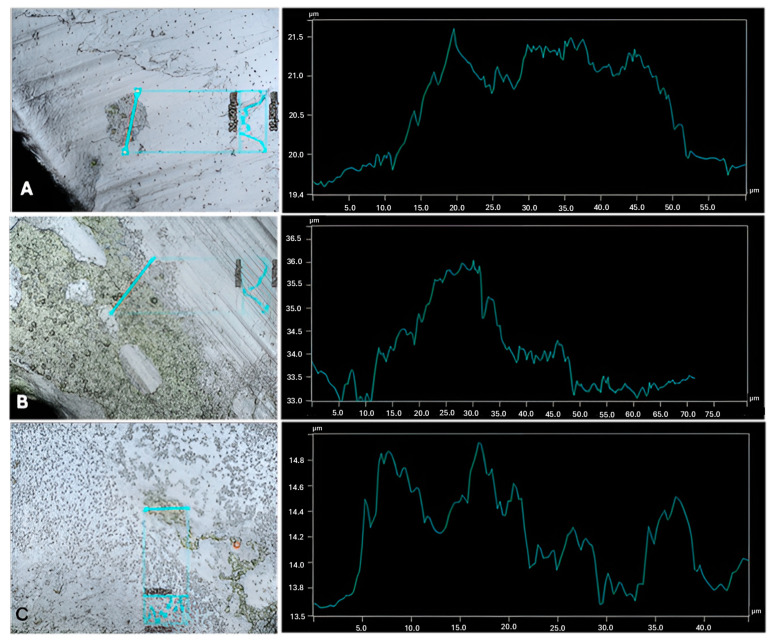
Biofilm profiles for C. vulgaris in co-cultivation with three different bacteria. (**A**) *C. vulgaris* w/*A. calcoaceticus*. (**B**) *C. vulgaris* w/*R. fascians*. (**C**) *C. vulgaris* w/*Leucobacter* sp. The imaging and the topography data were obtained using the profilometer.

**Figure 5:**
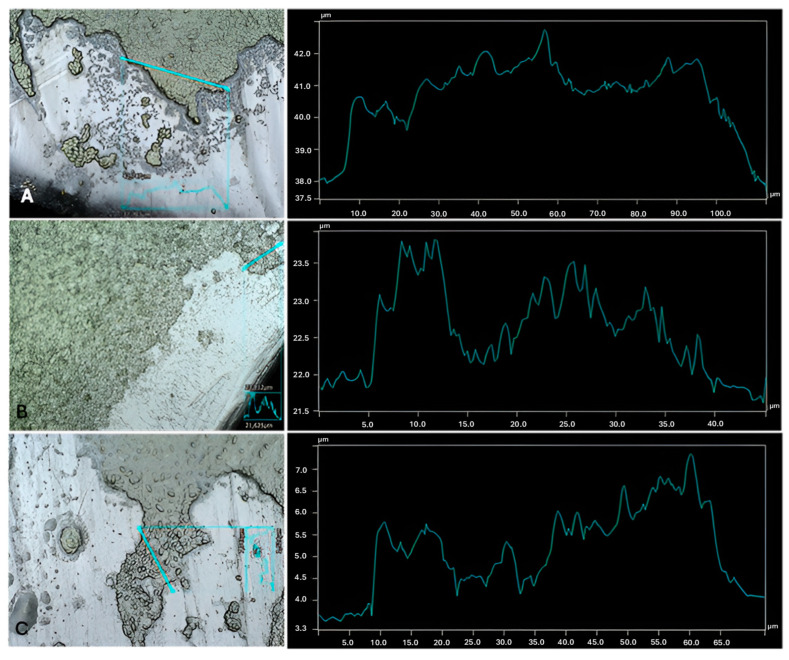
Biofilm profiles for *S. obliquus* in co-cultivation with the three bacteria. (**A**) *S. obliquus* w/*A. calcoaceticus.* (**B**) *S. obliquus* w/*R. fascians.* (**C**) *S. obliquus* w/*Leucobacter* sp. The imaging and the topography data were obtained using the profilometer.

**Figure 6:**
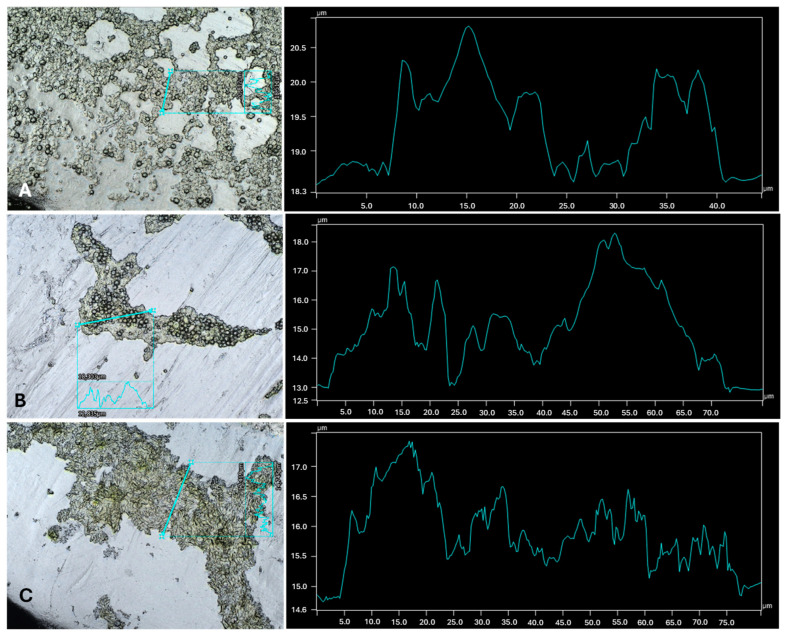
Biofilm profiles for axenic microalga. (**A**) *C. sorokiniana*. (**B**) *C. vulgaris*. (**C**) *S. obliquus*. The imaging and the topography data were obtained using the profilometer.

By looking at the dual-species biofilm profiles in Figure 3, Figure 4 and Figure 5 and their respective axenic cultures (Figure 6 and Figure S10), as well as Table 1, it is possible to observe how the different combinations of microalgae and bacteria cause structural alterations on the surface of the biofilm. Structure changes in biofilms can be affected by several factors, ranging from environmental conditions to the available nutrients [37]. However, in this work, the main differential factor is the combination of microorganisms, which is another significant factor affecting the structural build of the biofilm, EPS production [38]. The EPS production is different between microalgal and bacterial strains, and other stimulations can occur that can change the EPS release and potentially change the biofilm.

Interestingly, regarding the most considerable thickness detected on all combinations, *C. sorokiniana* developed a stronger biofilm in the presence of two bacteria (namely with *A. calcoaceticus* or with *R. fascians*, Table 1, *p* < 0.05), achieving the highest thickness with *A. calcoaceticus*, 4.51 ± 0.8 µm. Therefore, synergy in growth was observed for this combination of microorganisms in biofilm formation. However, this behavior detected via the consortium is not surprising because, as referred to before, the strain of *C. sorokiniana* selected for this work was isolated from a biofilm present in WW, implying previous contact with different microorganisms and leading to higher cellular growth via both *C. sorokiniana* and *A. calcoaceticus*. Although not statistically significant, the presence of *R. fascians* also promoted higher biofilm development (indicating cellular growth via both microorganisms), in contrast to the axenic culture, proposing a cellular growth stimulation of *C. sorokiniana* in the presence of bacteria (*p* > 0.05). The presence of bacteria can enhance the growth of microalgae, releasing growth-promoting hormones that may explain the higher biofilm development [4]. In particular, the *Acinetobacter* genus is related to phyto-stimulation through growth-promoting hormones, the production of siderophores, and phosphate solubilization. It can even inhibit the growth of bacteria and fungi that are harmful to microalgae [39,40]. Russel et al. [39] also reported the synergy between *Acinetobacter pittii* and *S. obliquus*. However, no stimulation on biofilm growth was found in this work regarding the same microalga co-cultivated with *A. calcoaceticus* (3.25 ± 0.3 µm for the axenic *S. obliquus* vs. 3.18 ± 0.6 µm for *S. obliquus* with *A. calcoaceticus*, Table 1, *p* > 0.05). The type of sessile cultivation plays an important role in the interaction between both types of microorganisms and the species and strains used for each consortium tested. This is evident by looking at the different combinations tested in this work. For example, *R. fascians* promoted the maximum thickness value, including the maximum average thickness and maximum biofilm peak values of all the bacteria co-cultivated with *C. vulgaris*. In contrast, the same bacterium thwarted biofilm development in the presence of *S. obliquus*. On the other hand, *S. obliquus* was not affected by the presence of *Leucobacter* sp., while the other two microalgae formed biofilms with the lowest values of maximum thickness (1.54 ± 0.2 µm for *C. vulgaris* with *Leucobacter* sp and 2.51 ± 0.3 µm for *C. sorokiniana* with *Leucobacter* sp), average thickness (1.03 ± 0.1 µm for *C. vulgaris* with *Leucobacter* sp and 2.09 ± 0.4 µm for *C. sorokiniana* with *Leucobacter* sp.), and most prominent peak (0.89 ± 0.2 µm for *C. vulgaris* with *Leucobacter* sp. and 1.46 ± 0.3 µm for *C. sorokiniana* with *Leucobacter* sp.) in co-cultivation with the same bacterium. Previous work testing *Leucobacter* sp. with *C. vulgaris* also revealed an antagonistic effect in cellular growth and nutrient removal when co-cultivated in suspension, indicating that this bacterium may not be the most favorable microorganism to introduce when the aim is to enhance the growth and bioremediation effects of microalgae [14]. Sousa et al. [41] also demonstrated antagonistic effects in the growth of *C. vulgaris* in the presence of *Helicobacter* sp., even by introducing higher concentrations of microalgal cells. This study observed the lowest biofilm development for all axenic bacterial biofilms, with the highest thickness detected in the axenic *R. fascians* biofilm (Table 1, *p* < 0.05). From these data and the biofilm profile pictures presented in Supplementary Materials, axenic bacterial growth was never observed on the surface of the K5 carriers, an effect related to the medium composition, which is not the most favorable for bacteria growth [42]. However, in the dual-species biofilms, bacteria favored biofilm development and the growth promotion of both microorganisms. In general, the use of profilometry yielded a detailed qualitative analysis of the biofilm development on the proposed carriers.

## 4. Conclusions

Higher biofilm development was detected on the consortia containing *C. sorokiniana* and *S. obliquus*, with some bacterial clusters being detected in the presence of microalgae. Also, *C. vulgaris*, axenic or in co-cultivation with bacteria, appears to cover less surface area than the biofilms formed by the other two microalgae. The highest values of thickness and the most prominent peak of biofilm measured were obtained in the *C. sorokiniana* with *A. calcoaceticus* consortium, while *Leucobacter* sp. had a negative impact on the biofilm development of *C. vulgaris* and *C. sorokiniana*. On the other hand, *S. obliquus* was not impacted by the presence of *Leucobacter* sp. Also, possibly since *C. sorokiniana* was previously isolated from a WW environment, the biofilm thickness involving this strain was higher in all cases where bacteria were present. All the data presented in this work show that *C. sorokiniana* was the microalga better adapted for co-cultivation with bacteria in WW. On the other hand, *A. calcoaceticus* and *R. fascians* were the bacteria that better provided the conditions for developing a dual-species biofilm for WW treatment. Therefore, the biofilm consortia that is more promising for growth in WW with K5 carriers is *C. sorokiniana* in co-cultivation with *A. calcoaceticus*. In general, this work shows that the selection of the microalgae/bacteria species will impact the biofilm formation ability, including the potential to form a biofilm and the sessile structure. Profilometry can be a viable measurement technique for biofilms on different surfaces, as future applications can expand by using other species and materials. Future evaluations will need to consider the polishing capacity of the biofilms tested here to fully evaluate the efficiency of the consortia, as well as introduce real WW to track the development of the consortia in the presence of other microorganisms and contaminants.

## Figures and Tables

**Table 1:** Biofilm parameters were measured for the axenic and co-cultivated cultures grown in the K5 carriers. Statistical differences were subjected to an ANOVA, followed by the Tukey–Kramer multiple comparison method. The mean values with different letters differ significantly (*p* < 0.05).

Cultures	Highest Thickness (µm)	Average Thickness (µm)	Biggest Peak (µm)
*C. vulgaris*	3.06 ± 0.9 ^a^	2.42 ± 0.7 ^a^	1.79 ± 0.7 ^a,b^
*C. vulgaris* with *A. calcoaceticus*	1.91 ± 0.1 ^a,b^	1.20 ± 0.1 ^b^	0.95 ± 0.1 ^a^
*C. vulgaris* with *R. fascians*	2.29 ± 0.4 ^a^	1.37 ± 0.3 ^b^	1.27 ± 0.3 ^a^
*C. vulgaris* with *Leucobacter* sp.	1.54 ± 0.2 ^b^	1.03 ± 0.1 ^b^	0.89 ± 0.2 ^c^
*C. sorokiniana*	2.55 ± 0.7 ^a^	1.07 ± 0.1 ^b^	1.21 ± 0.3 ^a^
*C. sorokiniana* with *A. calcoaceticus*	4.51 ± 0.8 ^c^	2.80 ± 0.9 ^a^	2.36 ± 0.5 ^a,b^
*C. sorokiniana* with *R. fascians*	3.44 ± 0.5 ^a,c^	2.39 ± 0.6 ^a^	2.14 ± 0.5 ^a,b^
*C. sorokiniana* with *Leucobacter* sp.	2.51 ± 0.3 ^a^	2.09 ± 0.4 ^a^	1.46 ± 0.3 ^a^
*S. obliquus*	3.25 ± 0.3 ^a,c^	1.93 ± 0.3 ^a^	1.72 ± 0.2 ^a^
*S. obliquus* with *A. calcoaceticus*	3.18 ± 0.6 ^a,c^	1.58 ± 0.5 ^a^	1.59 ± 0.2 ^a^
*S. obliquus* with *R. fascians*	1.78 ± 0.3 ^b^	1.38 ± 0.2 ^a^	1.12 ± 0.2 ^a^
*S. obliquus* with *Leucobacter* sp.	3.15 ± 0.3 ^a,c^	2.21 ± 0.2 ^a^	1.44 ± 0.2 ^a^
*A. calcoaceticus*	0.61 ± 0.2 ^d^	0.67 ± 0.4 ^c^	0.43 ± 0.2 ^d^
*R. fascians*	1.01 ± 0.2 ^e^	0.71 ± 0.1 ^c^	0.62 ± 0.1 ^d^
*Leucobacter* sp.	0.43 ± 0.1 ^f^	0.32 ± 0.1 ^d^	0.27 ± 0.1 ^e^

## Data Availability

The original contributions presented in this study are included in the article/Supplementary Materials. Further inquiries can be directed to the corresponding authors.

## Supplementary Materials

The following supporting information can be downloaded at https://www.mdpi.com/article/10.3390/microorganisms13051060/s1, Figures S1–S8—SEM images of the remaining microalgae–bacteria combinations; Figure S9—Live photographs taken at different magnifications through a profilometer; Figure S10—Biofilm profile of axenic bacteria.

## Abbreviations

The following abbreviations are used in this manuscript:

WW	Wastewater
WWTP	Wastewater Treatment Plant
SEM	Scanning Electron Microscopy
EPS	Extracellular Polymeric Substance
CEC	Contaminant of Emerging Concern
RAB	Rotating Algal Bioreactor
OECD	Organization for Economic Co-operation and Development
TSA	Tryptic Soy Agar
TSB	Tryptic Soy Broth
HDPE	High-Density Polyethylene
