# Supplementary material for: Qualitative Assessment of Microalgae–Bacteria Biofilm Development on K5 Carriers: Photoheterotrophic Growth in Wastewater
Source: Microorganisms. 2025 May 2;13(5):1060. doi: 10.3390/microorganisms13051060 (PMC12113768; doi:10.3390/microorganisms13051060)
Supplement: Supplementary file 1 [file microorganisms-13-01060-s001.zip › microorganisms-3545107-supplementary.pdf]

Supplementary Material

Qualitative Assessment of Microalgae–Bacteria Biofilm Development on K5 Carriers: Photoheterotrophic Growth in Wastewater

Henrique Sousa [1,2], Kerry A. Kinney [3], Cátia A. Sousa [1,2,4,5,*] and Manuel Simões [1,2,*]

[1] LEPABE—Laboratory for Process Engineering, Environment, Biotechnology and Energy, Faculty of Engineering, University of Porto, Rua Dr. Roberto Frias, 4200-465 Porto, Portugal
[2] ALiCE—Associate Laboratory in Chemical Engineering, Faculty of Engineering, University of Porto, Rua Dr. Roberto Frias, 4200-465 Porto, Portugal
[3] Architectural, and Environmental Engineering, Department of Civil, University of Texas, 301E E Dean Keeton St. c1700, Austin, TX 78712, USA
[4] ISEP/P.PORTO, School of Engineering, Polytechnic of Porto, Rua Dr. António Bernardino de Almeida, 431, 4249-015 Porto, Portugal
[5] CIETI, Center for Innovation in Engineering and Industrial Technology, School of Engineering, Polytechnic of Porto, Rua Dr. António Bernardino de Almeida, 431, 4249-015 Porto, Portugal
* Correspondence: fas@isep.ipp.pt (C.A.S.); mvs@fe.up.pt (M.S.); Tel.: +351-22-834-0500 (C.A.S.); +351-22-508-2262 (M.S.)

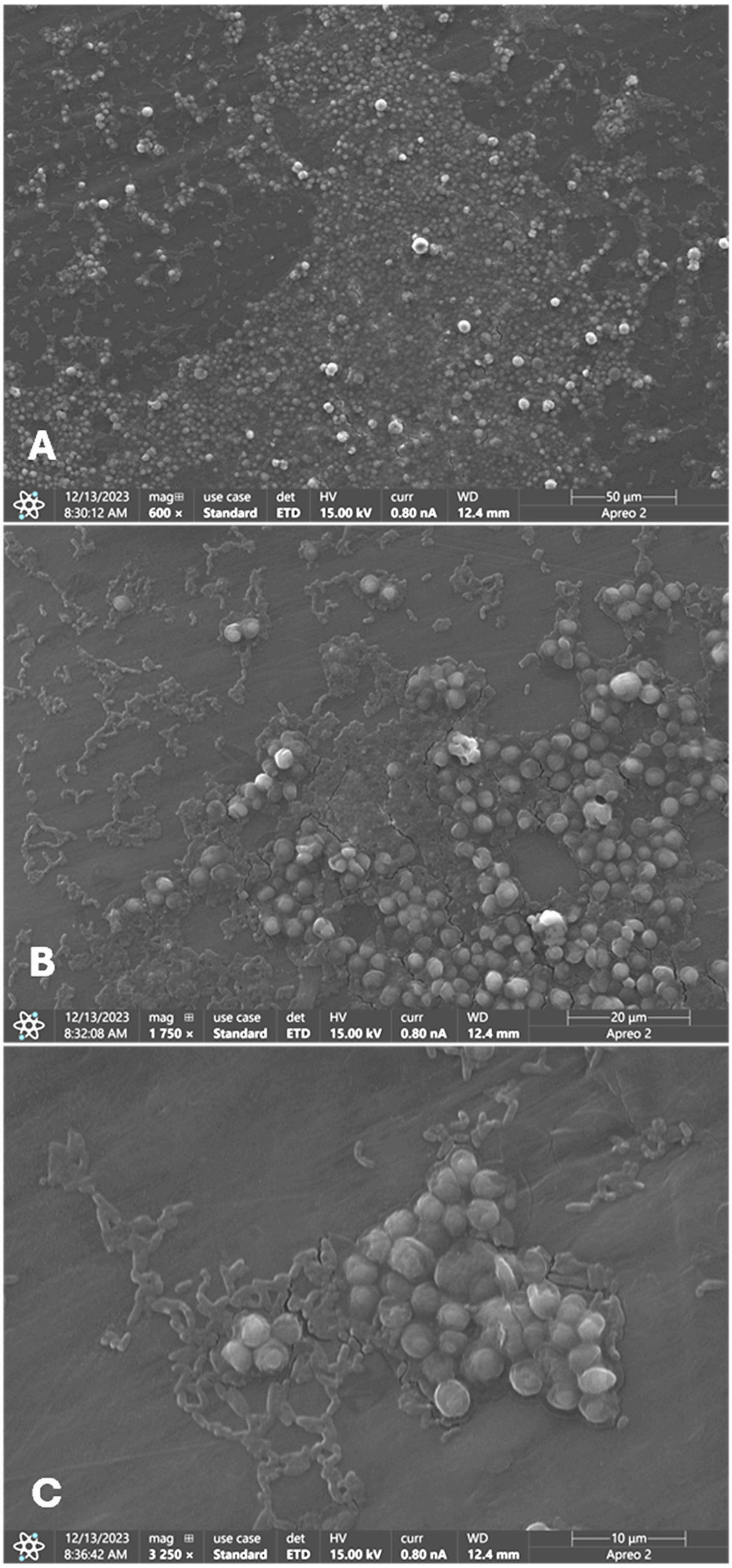

Figure SM.1 - SEM images of *C. sorokiniana* w/ *R. fascians* biofilm at different magnifications. A- 600×. B – 1750×. C – 3250×.

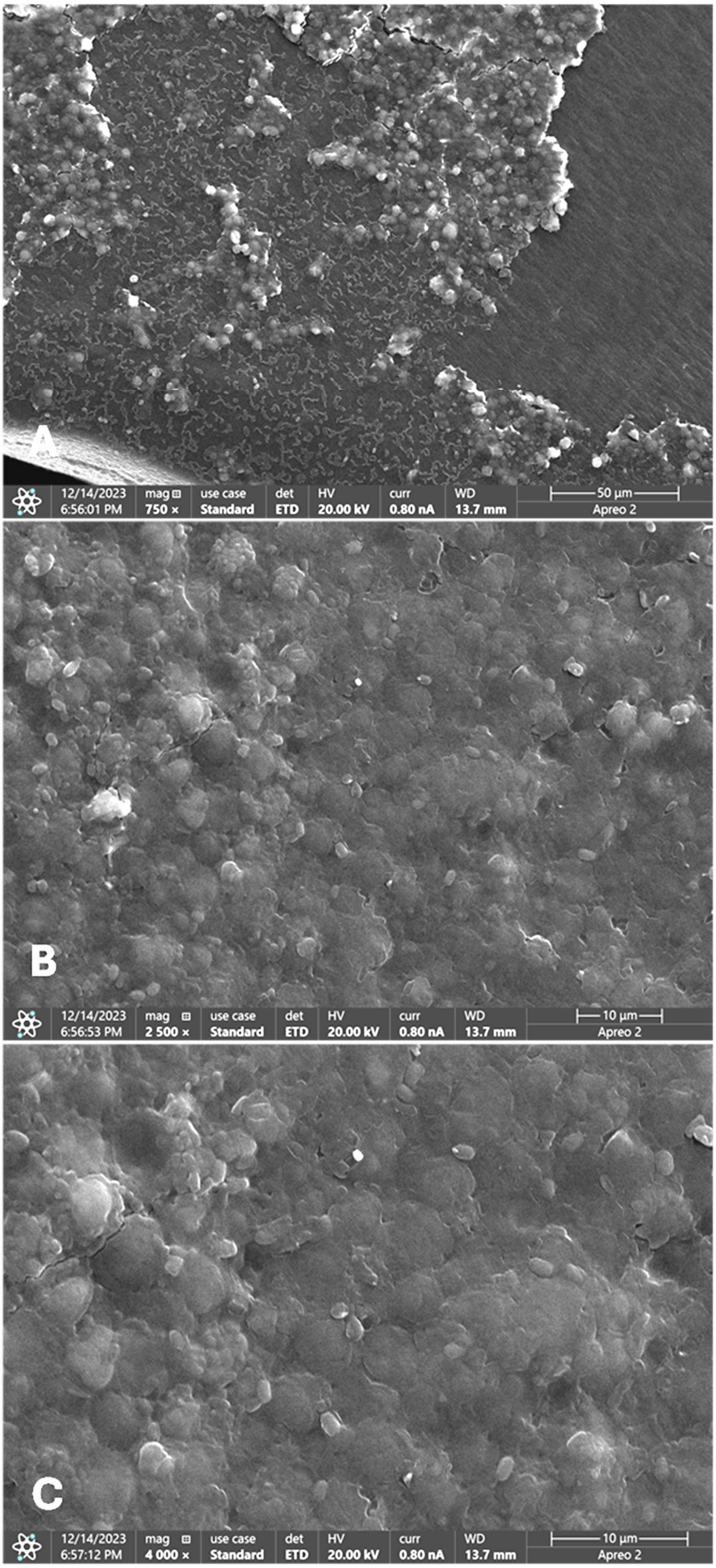

Figure SM.2 - SEM images of *C. sorokiniana* w/ *Leucobacter* sp. biofilm at different magnifications. A- 750×. B – 2500×. C – 4000×.

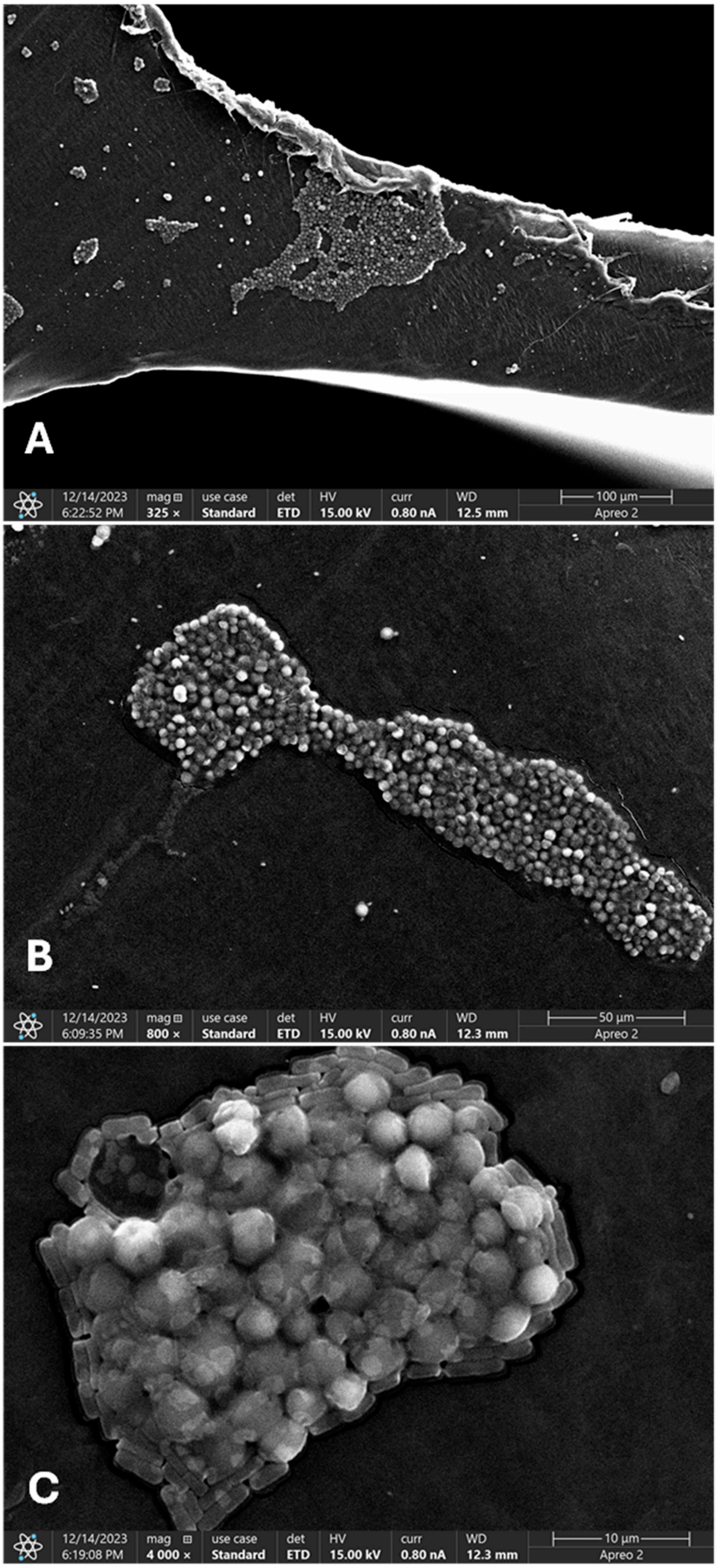

Figure SM.3 - SEM images of *C. vulgaris* w/ *A. calcoaceticus* biofilm at different magnifications. A- 325×. B – 800×. C – 4000×.

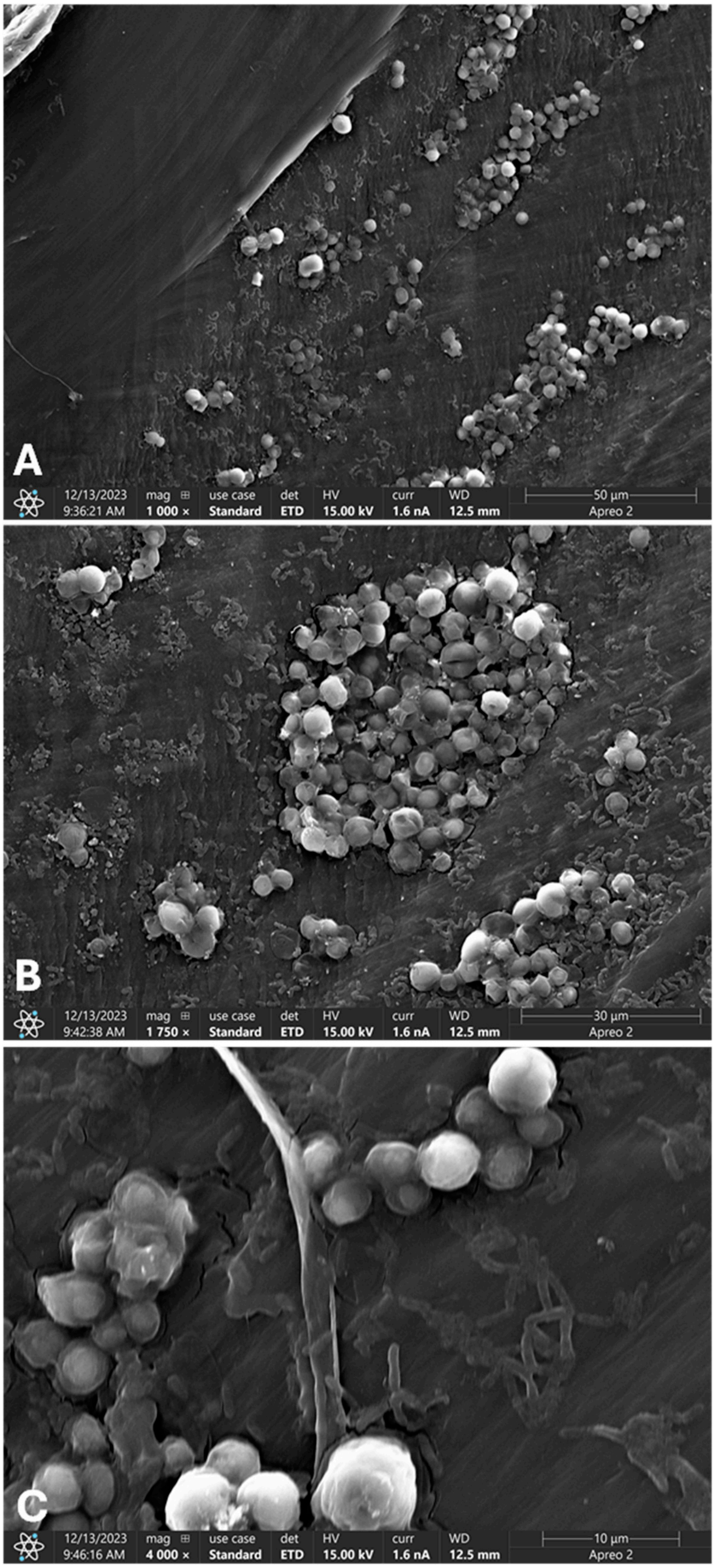

Figure SM.4 - SEM images of *C. vulgaris* w/ *R. fascians* biofilm at different magnifications. A-1000×. B – 1750×. C – 4000×.

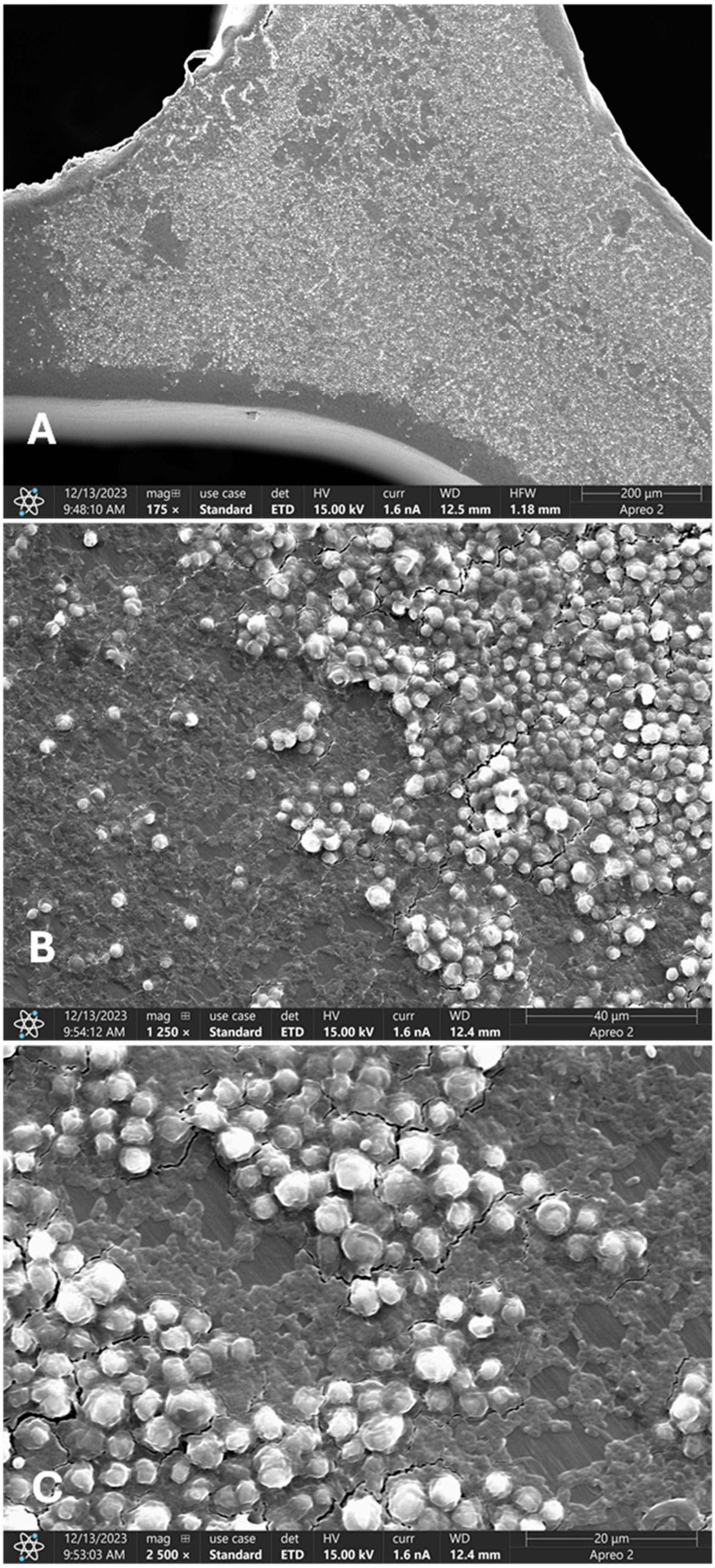

Figure SM.5 - SEM images of *C. vulgaris* w/ *Leucobacter* sp. biofilm at different magnifications. A- 175×. B – 1250×. C – 2500×.

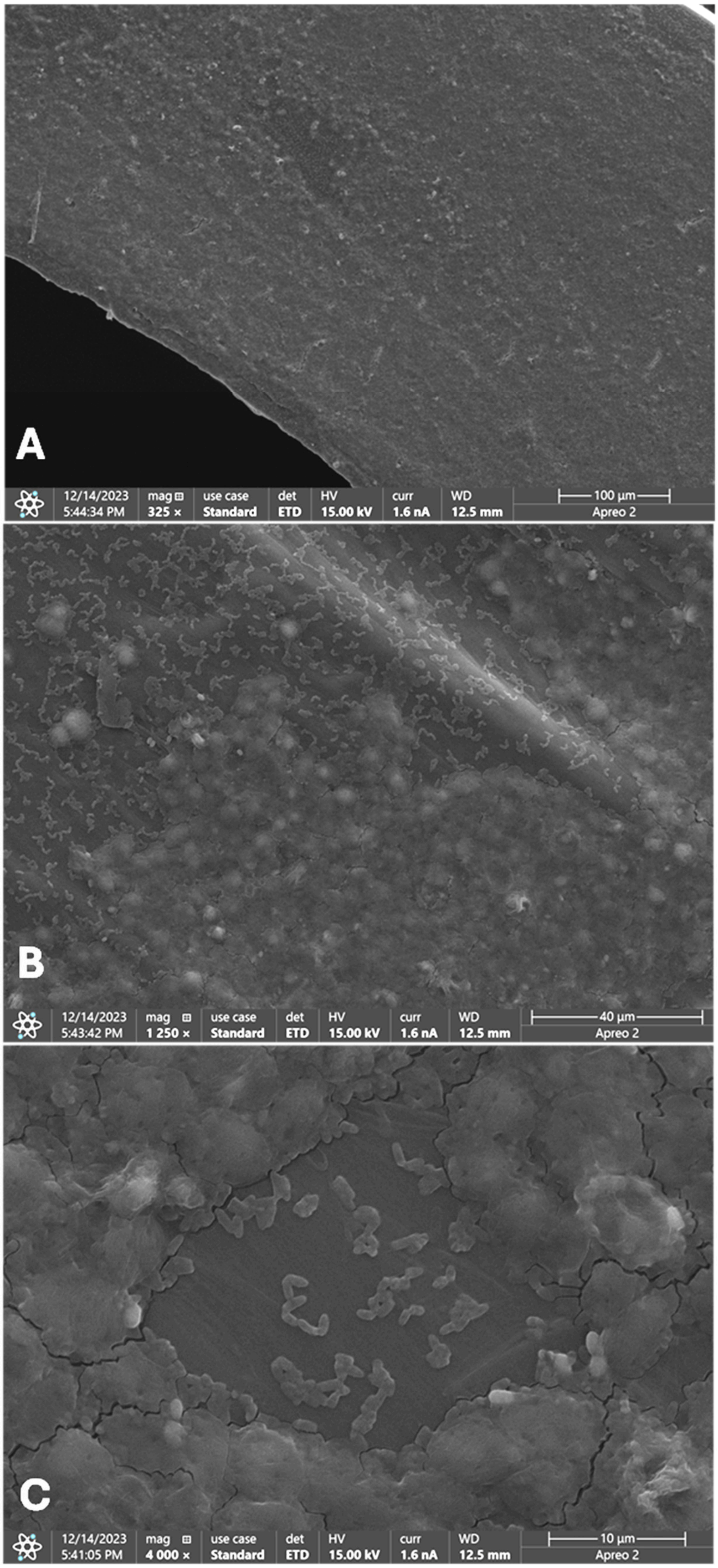

Figure SM.6 - SEM images of *S. obliquus* w/ *A. calcoaceticus* biofilm at different magnifications. A- 325×. B – 1250×. C – 4000×.

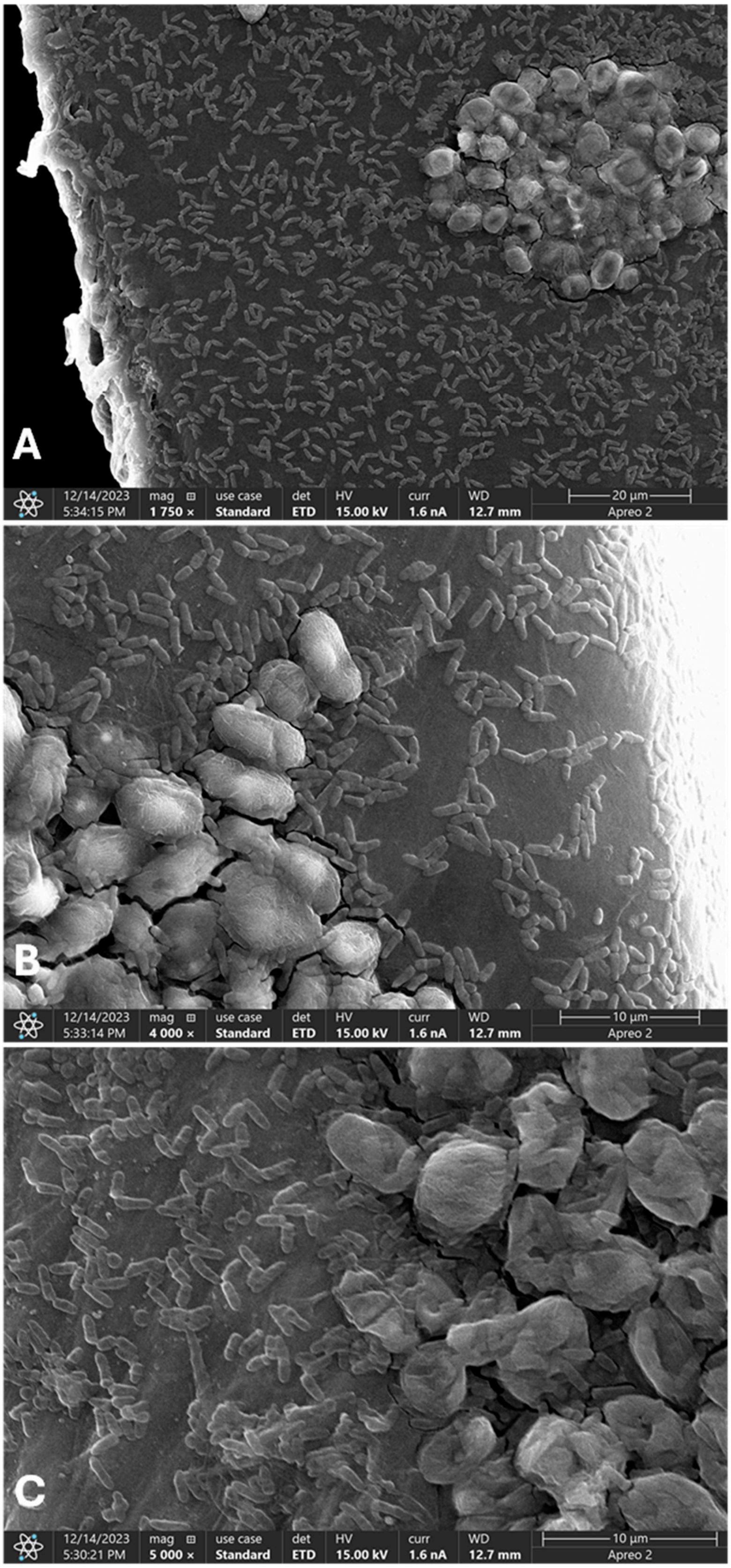

Figure SM.7 - SEM images of *S. obliquus* w/ *R. fascians* biofilm at different magnifications. A- 1750×. B – 4000×. C – 5000×.

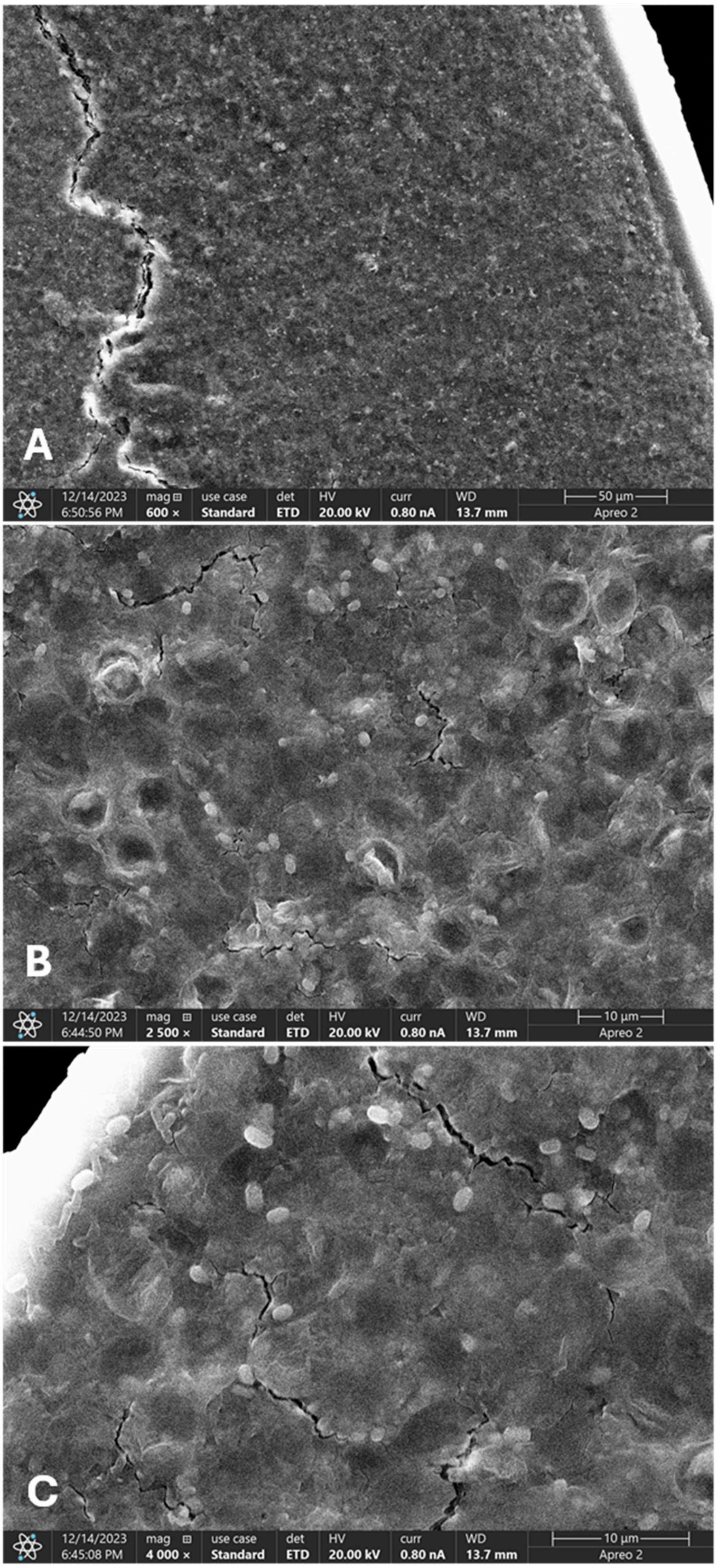

Figure SM.8 - SEM images of *S. obliquus* w/ *Leucobacter* sp. biofilm at different magnifications. A- 600×. B – 2500×. C – 4000×.

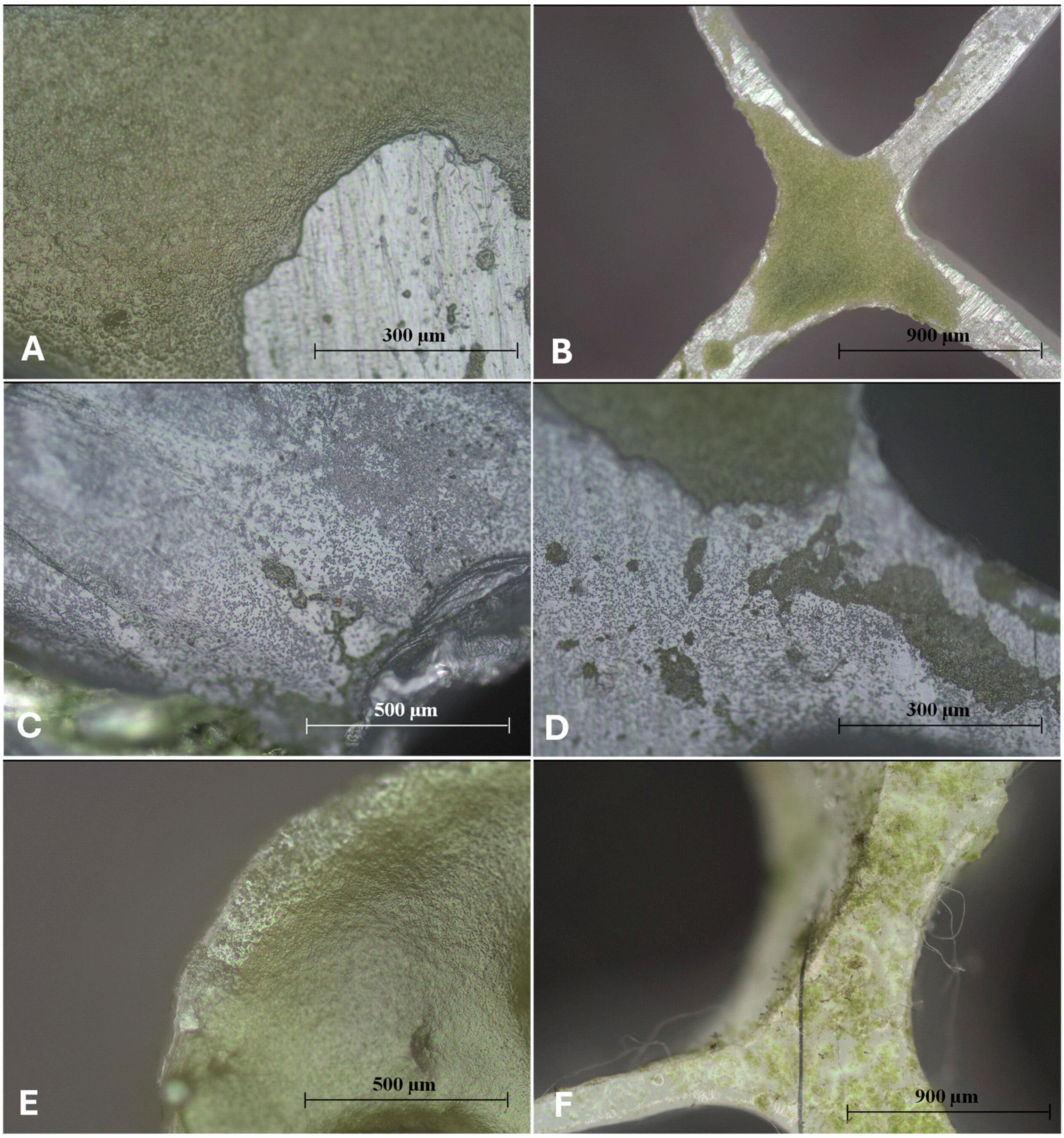

Figure SM.9 - Photographs taken at different magnifications through a profilometer for biofilm assessment. A – *S. obliquus* w/ *Leucobacter* sp. B – *S. obliquus* w/ *R. fascians*. C – *C. vulgaris* w/ *Leucobacter* sp. D – *S. obliquus* w/ *A. calcoaceticus*. E – Axenic *C. sorokiniana*. F – Fungal contamination.

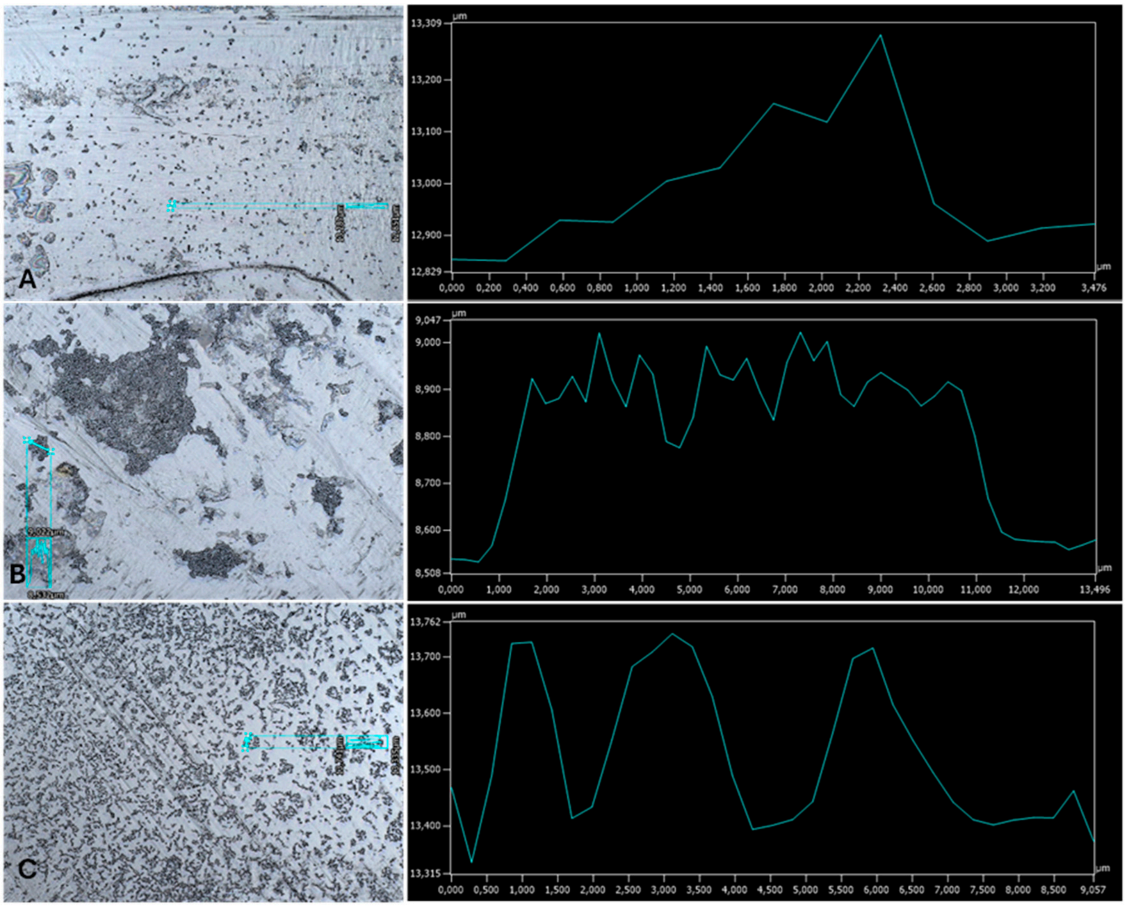

Figure SM.10 - Biofilm profile of axenic bacteria. A – *A. calcoaceticus*. B – *R. fascians*. C – *Leucobacter* sp.
